# Food aversion during pregnancy and its association with nutritional status of pregnant women in Boricha Woreda, Sidama Regional State, Southern Ethiopia, 2019. A community based mixed crossectional study design

**DOI:** 10.1186/s12978-021-01258-w

**Published:** 2021-10-18

**Authors:** Abel Yalew, Wondwosen Tekle Silasie, Anchamo Anato, Anteneh Fikrie

**Affiliations:** 1 Abel Medium Clinic, Gondar, Ethiopia; 2 School of Public Health, College of Medicine and Health Sciences, Hawassa University, Hawassa, Ethiopia; 3 School of Nutrition, Food Science and Technology, College of Agriculture, Hawassa University, Hawassa, Ethiopia; 4 School of Public Health, Institute of Health, Bule Hora University, 144, Hagere Maryam, Ethiopia

**Keywords:** Pregnancy, Food aversions, Nutritional status, Boricha Woreda, Southern Ethiopia

## Abstract

**Background:**

Despite high prevalence, food aversions are closely linked to the dietary intake of pregnant women. Thus, understanding this behavior is important in addressing the issue of maternal nutrition. Therefore, the aim of this study is to provide information on the prevalence and associated factors of food aversion and its relationship with the nutritional status of pregnant women in Boricha Woreda, Sidama Regional state, Southern Ethiopia, 2019.

**Methods:**

A community based mixed cross sectional study was conducted among 505 randomly selected pregnant mothers at Boricha Woreda, Southern Ethiopia from June 1–20, 2019. Pre-tested and structured face-to-face interview questionnaire and focus group discussion guide were used to collect quantitative and qualitative data respectively. The quantitative data were cleaned, coded and entered into Epi Info version 7.1.4.0 and then exported to SPSS IBM version 20 for further analysis. The qualitative data were analyzed manually using a content analysis.The bi-variable and multivariable logistic regression was used to identify the possible factors of food aversion. AOR with the respective 95% CIs was used to declare statistical significance.

**Results:**

Nearly, seven-in-ten (69.2%) of the pregnant women were averted of at least one food. Cereal (45.9%) and enset (44.2%) were averted by majority of the participants. The mean (± SD) MUAC measurement was 22.7 (± 2.4) cm. Pregnant women of age group of 24–28 [AOR = 3.04, 95% CI (1.72–5.35)] and 29–33 years [AOR = 2.00, 95% CI (1.02–3.92)], nausea during [AOR = 1.77, 95% CI (1.16–2.70)] and having additional meal [AOR = 1.68, 95% CI (1.02–2.75)] were significantly associated with food aversion. Maternal nutritional status and food aversion was sstatistically significant (*p*-value < 0.001).

**Conclusion:**

High prevalence of food aversions (69.2%) and under nutrition (34.6%) among pregnant women is found. Therefore, the Woreda Health Office needs to intensify the integration of maternal nutrition into ANC services and training of health providers as well as critical appraisal of health extension workers should also be considered.

## Plain language summary

Pregnancy is a complex and absolutely important period in women’s life. Thus, in order to sustain a successful pregnancy, a mother experiences a number of physiological and behavioral changes. Notably, food aversions are the number one changes experienced by pregnant women. Food aversions are characterized by the repulsion and avoidance of particular foods and the most common aversions are nonalcoholic caffeinated beverages, meat, fish, poultry and eggs. Therefore, the aim of this study was to determine the magnitude and its association with nutritional status of Pregnant Women in order to provide concrete scientific evidence for the concerned stakeholders.

A community based mixed cross-sectional study was conducted among 505 pregnant mothers selected from five randomly selected kebeles of Boricha Woreda. Pregnant women in the age range of 19 to 49 years and who were apparently healthy were interviewed. Four hundred ninety seven participants were interviewed voluntarily with a response rate of 98.4%. The mean (± SD) age of the women was 22.3 (± 5.5) years. Pregnant women who were averted at least one food were 344(69.2%); where cereal (45.9%) and enset (44.2%) were averted by the majority of the participants. Hence pregnant women who practice food aversion had higher likely to be a malnourished. Pregnant women who were less than thirty three years of age, who had nausea, and ate additional meal were more urged to avert a particular foods. Whereas, women with better nutritional status had less food aversion practice.

In conclusion; despite a pregnant woman requires a healthy diet embedded with adequate intake of energy, protein, vitamins and minerals to meet maternal and fetal needs, the magnitude of food aversion and maternal under nutrition in the study area was high.

## Background

Pregnancy is a complex and absolutely important period in women’s life. Its physiology is of great biological and nutritional importance [1]. Unsurprisingly, pregnant mother sustain innumerable physiological and behavioral changes during the period of their pregnancy. Changes that will occur during the pregnancy period could lead to poor maternal and fetal health outcomes [2]. Nearly all pregnant mothers’ experience at least one food aversion throughout the period of pregnancy [3]. It is necessary to differentiate food aversion from avoidance. The later one is a condition characterized by avoiding certain types of food, having restricted intake in terms of overall amount eaten, or both as some the foods might be harmful to the women or the fetus because of the way they are prepared or because of germs or chemicals they contain [1]. Food aversions are characterized by sudden appearance with strong intensity of the repulsion toward foods with strong smells [4] and usually emerged at the end of the first trimester and intensify during the second trimester and gradually became diminished [5]. The prevalence of food aversions occurrings globally varies from 50 to 90%, being less common in European populations and more common in the African continent [1]. Likewise, in Tanzania 70.1% [6], in Nigeria 57.2% [7] and in Southern Ethiopia the prevalence of 65% to 67.8% were reported [8, 9].Pertainng to food types averted, western women were highly averted a protein-rich foods of animal origin whereas cereals, tea, stiff porridge and vegetables were the most frequently avoided foods by Africa and Asian pregnant women [1, 6].

The causes and consequences of food aversion is still basically unknown, but is hypothesized to be multifactorial [1]. Of the numerous hypotheses suggested to explain the occurrence of food aversion; the most typical are maternal–fetal protection, preventive of the metabolic syndrome, ensuring adequate nutrition, a change in olfactory and taste sensitivity, psychological impacts, hormonal effects and the influence of culture [1, 10–12]. Hormonal change during pregnancy is played a huge role in food aversions [3]. Nausea and vomiting are also mentioned as the principal factors for the development of food aversions [13, 14]. A study found a significant positive correlation between the week of onset of nausea and aversions. Similar study reported that taste aversion learning was found to be one of the mechanism for the development of food aversions during pregnancy [10]. Foods expected to exacerbate the challenges of pregnancy were reported to be predominatly averted [15]. Some studies corroborated the hypotheses that food aversions are supposed to be occurred so as to protect the fetus from external toxins or pathogen [11, 16].

Remarkably, despite the high prevalence of food aversion and its significant impact on the health of fetus and nutritional status of pregnant women, there is scarce research done on food aversion and its association with nutritional status of pregnant women globally as well as in the study area. As food aversions are closely linked to the dietary intake of pregnant women, understanding this behaviour is important in addressing the issue of maternal nutrition. Therefore, the aim of this study is to provide information on the prevalence and associated factors of food aversion, the relationship with the nutritional status of pregnant women and exploring the notion of the community about food aversion during pregnancy in Boricha Woreda, Sidama Regional State, Southern Ethiopia, 2019.

## Methods and materials

### Study setting

The study was conducted in Boricha District, Southern Ethiopia. The district is one of the 33 districts of Sidama regional state. It is located 32 km far from the Regional City, Hawassa and 297 km far southwest of Addis Ababa, the capital City of Ethiopia. The climatic condition of the district is ‘kola’, with an altitude of 1400 m above sea level and has an average temperature of 29°c. As the data from the Boricha district health office shows, the woreda has 14 kebeles (1 urban and 13 rural kebeles) with a total population of 121,648, of which 60,216 male wheras 61 are, 432 are females. The estimated number of pregnant women in the district is 4209. Regarding the heath infrastructure of the district, there are 3 governmental Health Centers, 13 Health Posts and 1 District Hospital. Sidama Regional State Health Departement Bureau report revelead that, Boricha District categorized under hot spot for malnutrition. The staple foods in the distric are maize and ‘Enset’ [17].

#### Study design and period

A community based cross sectional study design supplimented with a phenomenological qualitative approach was employed at Boricha Woreda, Sidama Regional State, Southern Ethiopia from June 1–20, 2019.

#### Source, study population and eligiblity criteria

All pregnant women who lived in Boricha Woreda were the source population of the study whereas pregnant women in a randomly selected five kebeles were our study populations. Pregnant women in the age range of 19 to 49 years and who were apparently healthy were included in this study. Whereas, pregnant women who lived less than six months in Boricha Woreda and Pregnant women who have chronic disease such as HIV/AIDS, TB and acute infectious disease such as malaria, typhoid fever and typhus were excluded from the study.

#### Sample size determination and sampling procedure

The sample size for first objective was determined using single population proportion formula based on the following assumptions: Proportion of food aversion (65%) a study conducted at Southern Ethiopia [8], 95% Confidence interval 5% margin of error, design effect of 1.5 and 10% for compensation of non-response rate. Then, the minimum calculated sample size became 576. However, the number of total pregnant women in the district were less than 10,000 (N = 4,209), we considered the population correction formula to get the appropriate representative sample size. Consequently, the final sample size after correction became 505. A two stage sampling technique was employed to select the study participants. In the first stage, the lists of Kebeles found in the district were gathered from the Boricha district Health Office. Then, the kebeles were stratified into rural (13 kebeles) and urban (1 kebele). Subsequently, the only 1 urban kebele and 4 rural kebeles were selected randomly and the sample sizes allocated to each kebele based on population proportion allocation. Ultimately, simple random sampling technique using random numbers generated by OpenEpi software was used to select 505 study participants. For the qualitative study we used five Focus Group Discussions (FGDs). Study participats for FGDs were identified and invited with the assistance of rural health extension workers working at the selected kebele.

#### Data collection and quality assurance

Five diploma holder nurses and two BSc holder Public Health Officers were recruited as data collectors and supervisors respectively. Two days training was given for both data collectors and supervisors on research ethical principles and data collection techniques and procedures and on the basic techniques of Mid Upper Arm Circumference (MUAC) measurement record. Furthermore, the investigators also assessed the quality of the data during the data enrty and analysis stage to verify the completeness of the collected data. In order to maintain the validity and reliability of the study the socio-demographic and economic characteristics, meal pattern and maternal health information were collected by a pretested, structured and validated face-to-face-interviewer administered questionnaire which were adapted from previous similar studies [8, 9, 15, 18]. In addition to this, the adapted questionnaires were sent to the nutritionist for further validation. Regarding food aversion, nausea and vomiting measures. We used structured and semi- structured face-to-face-interviewer administered questionnaire. The study participants were inquired to report if they had incidents of nausea, vomiting and aversive foods during their most recent pregnancy. Further, the women were also asked to list aversive food types. The severity of food aversion, nausea and vomiting were also assessed by asking: the duration of feelings of nauseated, whether or not the women considered terminating pregnancy due to your nausea and food aversion, whether or not ever considered not having more children due of your nausea and aversive food. Additionly, for the assessment of nutritional status the pregnant women MUAC measuring tape, a non stretchable tape calibrated to 0.1 cm was taken. According to the sphere project minimum standards in food security, nutrition and food aid cut-off point; a pregnant women with MUAC < 23.0 cm (undernourished) and pregnant women with MUAC ≥ 23.0 cm (well-nourished) [19]. Whereas the qualitative data were collected through FGDs, using an open ended focus group discussion guide and the data were recorded by audio taped.

#### Data processing and analysis for quantitative data

The data were thoroughly checked, cleaned, coded and then entered into Epi Info version 7.1.4.0 and exported to the Statistical Package for Social Science (SPSS) version 20 for analysis. Descriptive analysis was ran to assess missing values and presence of outliers. Mean and Standared deviation were used to summarize the numerical variables and the data were presented using frequency tables, figures and charts. Principal component analysis (PCA) was computed for constructing the wealth index of the study participants. The bi-variable and multivariable logistic regression was used to identify the possible factors of food aversion. A variable with *p*-value ≤ 0.25 during bivariate analysis were further entered into multivariate logistic regression to control the effect of confounding variables. Multi co-linearity was checked by Variance inflation factor. Finally, Adjusted Odds Ratio (AOR) and 95% Confidence Intervals (CIs) were used to declare statsticall significance. The qualitative data were analyzed manually using a qualitative content analysis method. First the data were transcribed in into the local language “Sidamic” and then translated in to English. After the completion of the translation the data were coded and categorized accordingly.

## Results

### Socio-demographic and economic characteristics of the study participants

From a total of 505 randomly selected pregnant women, 497 were interviewed voluntarly with a response rate of 98.4%. Nearly, four in five 389 (78.3%) of the respondents were rural residents. The mean (± SD) age of the women was 22.3 (± 5.5) years and the majority, 188 (37.8%), were in between 24 and 28 years of age. More than three fourth (396) of the study participants were Protestant religion followers. Nearly four in nine (223 and 225) of the study participants and their husbands had attended primary education respectively. The majority of the study participants and their husbands were house wives and farmers respectively. Regarding the wealth index of the study participants, the two extremities were almost equally represented with the poorest-to-richest ratio of 0.97 (Table 1).

**Table 1:** Socio-demographic characteristics of the pregnant women in Boricha woreda, Sidama Regional State, Southern Ethiopia, 2019

Variable (N = 497)	Frequency (N)	Percentage (%)
Residence		
Urban	108	21.7
Rural	389	78.3
Maternal age in years		
19–23	139	28
24–28	188	37.8
29–33	78	15.7
≥34	92	18.5
Religion		
Protestant	396	79.67
Orthodox	31	6.23
Muslim	70	14.1
Educational status women		
No formal education	149	30
Primary	223	44.9
Secondary	65	13.1
Above secondary	60	12.1
Educational status of husbands		
No formal education	88	17.7
Primary	225	45.3
Secondary	109	21.9
Above secondary	75	15.1
Occupation of women		
House wife	355	71.4
Student	22	4.4
Government employed	50	10.1
Merchant	58	11.7
Others	12	2.4
Occupation of husbands		
Government employed	68	13.7
Merchant	189	38
Farmer	197	39.6
Others	43	8.7
Wealth index		
Poorest	99	19.9
Poor	106	21.3
Middle	99	19.9
Rich	91	18.9
Richest	102	20.5

### Maternal health and anthropometric characteristics

One hundred twelve (22.5%) of the study participants were in their first pregnancy. Two hundred and seventy three (54.9%) of the study subjects were in their second trimester of pregnancy and 315 (63.4%) of the study participants reported that they have ANC follow up during the study period. Out of the total study participants 188 (37.8%) of the women didn’t experience nausea and/or vomiting during their current pregnancy but more than half, 282 (56.7%) of the women said they experience nausea during their current pregnancy. The average MUAC of the study participants was 22.7 ± 2.4 cm. More than one-third, 172 (34.6%) of the study participants was undernurished (MUAC < 23 cm) (Table 2).

**Table 2:** Maternal health and anthropometric characteristics of pregnant women in Boricha Woreda, Sidama Regional State, Southern Ethiopia, 2019

Variable (N = 497)	Frequency (N)	Percentage (%)
Trimester of pregnancy		
Second trimester	273	54.9
Third trimester	224	45.1
Attend antenatal care		
Yes	315	63.4
No	182	36.6
Parity		
Primiparous	112	22.5
Multiparous	385	77.5
Nausea and vomiting during pregnancy		
Nausea	282	56.7
Vomiting	148	29.8
Both nausea and vomiting	121	24.3
No	188	37.8
Nutritional status		
Well nourished (MUAC ≥ 23 cm)	325	65.4
Under nourished (MUAC < 23 cm)	172	34.6

### Meal pattern of the study participants

Out of the total study participants, 245 (49.3%) eat three times per day. Majority, 347 (69.8%) of the study subjects did not eat an additional meal during the period of their current pregnancy. Of those women who ate an additional meal during the period of their pregnancy 137 (91.3%) and 13 (8.7%) of them ate one and two additional meals per day respectively. According to meal skipping practice, 102 (20.5%) of the women skip at least one meal per day and lunch was the most frequently skipped meal, with the proportion of 67 (65.7%) followed by dinner, 35 (34.3%) (Table 3).

**Table 3:** Meal pattern of pregnant women in Boricha woreda, S Sidama Regional State, Southern Ethiopia, 2019

Variable (N = 497)	Frequency	Percentage (%)
Number of meal per day		
Two times	102	20.5
Three times	245	49.3
Four tmes	137	27.6
Five times	13	2.6
Skipping meal		
Yes	102	20.5
No	395	79.5
Type of meal skipped (n = 102)		
Lunch	67	65.7
Dinner	35	34.3
Having additional meal		
Yes	150	30.2
No	347	69.8
Number of additional meal (n = 150)		
One	137	91.3
Two	13	8.7

### Prevalence of food aversion of the study participants

Out of the total study participants, 344 (69.2% (95% CI: 67.2–71.2%)), had reported food aversion of at least one food during their current pregnancy. Out of 344 pregnant women who reported food aversion, nearly three-fourth, 252 (73.3%) of the participants, avert more than two kinds of food.Whereas, cereal and its products 45.9% and enset products 44.2% were found to be averted by majority of the women. Incontrary, Egg 1.7% and fish 2.3% were found to be the least food categories averted by the study participants. Regading the participants reasons for the aversion of food, the majority (35.8%) reported that they do not know the reason why they averted a specific food during their pregnancy. Whereas, 22.4 and 25.9% of women reported that, their reason of avoiding a certain kind of food was because of nausea and vomiting (Table 4).

**Table 4:** Reported food aversion of pregnant women in Boricha woreda, Sidama Regional State, Southern Ethiopia, 2019

Variable (N = 344)	Frequency (N)	Percentage (%)
Number of foods avoided		
1	92	26.7
≥2	252	73.3
Categories of foods		
Cereal and cereal products	158	45.9
Roots and tubers	50	14.5
Legumes and legume products	23	6.7
Vegetables	39	11.3
Fruits	59	17.2
Meat and meat products	18	5.2
Egg	6	1.7
Fish	8	2.3
Dairy products	31	9
Oils and fats	51	14.8
Coffee	48	14
Foods which has strong smell	48	14
Soft drinks	9	2.6
Sweets	12	3.5
Kocho/enset products	152	44.2
Reasons for food aversion		
Nausea and vomiting	77	22.4
Heart burn	89	25.9
Don’t know	123	35.8
Personal dislike of the food	23	6.9
Smell and test of the food	48	14
Having/eating frequently	31	9
Believe dislike by the fetus	32	9.3

In line with the finding of the quantitative part of the study, many of the pregnant women who had participated in the FGDs said “they do not know why pregnant women avoid or hate certain foods during their pregnancy”. However, most participants gives that nausea amd vomiting are the reason for food aversion occurred during pregnancy.

“I used to avoid foods which have strong smell and foods which have been cooked with butter during the first four months of pregnancy as they give me nausea and vomiting. But latter, just after I turned to my six months of pregnancy, I started to eat those foods that I used to avoid during the early stage of my pregnancy.”(An eight month 30 years old pregnant woman)

Similarly health concern of both the mother and the baby was given as a reason for food aversion practice during pregnancy. The participants of the FGDs believe that, pregnant women avoid certain foods as the woman’s body hated it because it is not good for the health of the baby and/or the mother’s health.

“I used to avoid meat during the first three months of pregnancy. I don’t know why I hated meat, but I do believe that may be my baby hates it or my body dislikes this particular food.”(A thirty seven years old woman who has three children and on her seven months of pregnancy)

### Factors associated with food aversion

 Pregnant women of age group of 24–28 and 29–33 of years were 3 and 2 times more likely to experience food aversion as compared to pregnant women of age ≥ 34 years of age [AOR = 3.04, 95% CI (1.72–5.35)] and [AOR = 2.00, 95% CI (1.02–3.92)] respectively. Pregnant women who experienced nausea during pregnancy were found to be 1.7 times more likely to have food aversion [AOR = 1.77, 95% CI (1.16–2.70)]. A pregnant women who ate additional meal was 1.7 times more likely to suffer from food aversion as compared to their counter parts [AOR = 1.68, 95% CI (1.02–2.75)]. Moreover wellnourished pregnant women were 62% times less likely of experiencing food aversion as compared to undernourished pregnant women [AOR = 0.38, 95% CI (0.23–0.62)] (Table 5).

**Table 5:** Factors associated with food aversion during pregnancy in Boricha woreda, Sidama Regional State, Southern Ethiopia, 2019

Variable		Food aversion		COR (95%CI)	AOR (95% CI)
		Yes N (%)	No N (%)		
Age	19–23	99 (71.2)	40 (28.2)	2.37 (1.36–4.10)	2.02 (0.87–4.66)
	24–28	145 (77.1)	43 (32.1)	3.22 (1.89–5.49)	3.04 (1.72–5.35)***
	29–33	53 (67.9)	25 (24.5)	2.03 (1.08–3.80)	2.00 (1.02–3.92)*
	≥34	47 (51.1)	45 (48.9)	1	1
Residence	Urban	74 (68.5)	34 (31.5)	0.95 (0.60–1.51)	0.64 (0.33–1.25)
	Rural	270 (69.4)	119 (30.6)	1	1
Educational status of women	No formal education	104 (69.8)	45 (30.2)	0.84 (0.43–1.64)	1.01(0.47–2.15)
	Primary	152(68.2)	71 (31.8)	0.77 (0.41–1.47)	0.91(0.44–1.86)
	Secondary	44 (67.7)	21 (32.3)	0.76 (0.35–1.65)	0.78 (0.34–1.76)
	Tertiary	44 (73.3)	16 (26.7)	1	1
Parity	Primi	84 (75)	28 (25)	1.44 (0.89–2.32)	1.38 (0.64–2.98)
	Multi	260 (67.5)	125 (32.5)	1	1
ANC	Yes	247 (73.7)	88 (26.3)	1.88(0.99–2.29)	–
	No	97 (59.9)	65 (40.1)	1	–
Nausea	Yes	212 (75)	70(25)	1.90(1.29–2.79)	1.77 (1.16–2.70)**
	No	132 (61.4)	83 (38.6)	1	1
Skipping meal	Yes	73 (71.6)	29(28.4)	1.15(0.71–1.86)	–
	No	271 (68.6)	124(31.4)	1	–
Additional meal	Yes	115 (76.7)	35(23.3)	1.69(1.09–2.62)	1.68 (1.02–2.75)*
	No	229 (66)	118(34)	1	1
Nutritional status	Well-nutrition	212 (63.2)	113 (34.8)	0.56(0.37–0.86)	0.38 (0.23,0.62)***
	Under nutrition	132 (76.7)	40 (23.3)	1	1

*significant at p-value < 0.05, **significant at *p*-value < 0.01, ***significant at *p*-value < 0.001

### Relationship between food aversion and nutritional status of pregnant women

After controlling for the potential counfounding variables, the study found that food aversion and nutritional status of pregnant women has a statistically significant association (*p*-value < 0.001). The prevalence of food aversion being highest among undernourished pregnant women. Hence pregnant women who practice food aversion are more likely to be malnourished (Table 5).

## Discussion

The prevalence of food aversion 69.2% (95% CI:67.2%-71.2%), found in this study is similar to the prevalence of food aversions reported globally by other researchers, ranged from 50 to 90% [1]. The result of this study is in line with similar other studies conducted in Ethiopia: Hadiya Zone [8] and Sidama Zone Dale Woreda [9]. Studies conducted elasewhere also concluded the same Tanzania [6], Nigeria [4] and Ecuador [14]. However, the finding of this study is inconsistent with five other studies found a prevalence of an interval in betwen 39%-57%,which are conducted out side Ethiopia [20].

Despite being the stable foods of the study area, cereal and its products and enset and enset products were the most frequently averted foods identified by this study. This finding is in line with the same studies conducted in Ethiopia: Hadiya Zone [8] and Sidama Zone [9]. However, the result of this study is not in accord with a study result conducted at Tanzania; where the vast proportion of pregnant women avoided meat and fish [6]. The observed variation might be due to the study participants difference in thier culture, tradition and socio-economic status as culture and traditions are highly tied into the preferences of food, and the chance of evolving food aversion [1]. In South India, “hot” foods items like papaya and “black” foods like naval, black grapes, and sesame are commonly avoided foods owing to the perception that they cause harm to the fetus [11]. Moreover, there is also an evidence that Ethiopian pregnant women crave for meat and its product rather than aversting it [8].

The result also supports the notion that aversion to commonly consumed foods is an inbuilt mechanism to diversify the types of foods consumed by avoiding monotonous diet [3]. Unsurprisingly, the high proportion of aversion to cereal, which contains a significant amount of phytate that reduce the bioavailability of zinc, iron and calcium, seen in this study supports the assumption that, pregnant women avoid foods that contain plant toxins/phytochemicals [21]. More importantly, it supports the hypothesis; “dietary aversions as preventive of the metabolic syndrome during pregnancy”. Aversions to these carbohydrate-rich foods were possibly a mechanism to prevent the gestational metabolic syndrome [1].

A change in olifactory and taste sensitivity, which could result in nausea, is considered as possible factor that arbitrate the development of food aversion in pregnant women [1]. Our study also found that pregnant women who experienced nausea were found to be 1.7 times more likely to have food aversion than those who were not experienced nausea during their pregnancy [AOR = 1.77, 95% CI (1.16–2.70)]. This result is in line with the report of other similar studies conducted in different areas [3, 20]. Likewise, the finding of our result supports the assumptions of anthropologists who have suggested that aversion is mainly evolved due to nausea (morning sickness) [3]. This might be explained that; the presence of nausea help the women to expel offending foods containing potentially dangerous substances as the protection mechanism of both for her health and her baby’s health [21].

The WHO recommended at least one additional meal during the period of pregnancy [22]. Similarly, a systematic review on maternal diet during pregnancy revealed that; increased consumption of food during pregnancy was thought to “strengthen the child's body” [23]. However, only about 31.3% of study participants were found to eat an additional meal. This is comparable to the report of the study done in Ethiopia; Sidama Zone Dale Woreda [9]. The observed comparability is probably due to the similarity of the study area, Sidama Zone. On the other hand our study found significant association between food aversion and having additional meal. Thus, pregnant mothers who ate additional meal were 1.70 times more likely of avoiding food than those who had not ate additional meal [AOR = 1.68, 95% CI (1.02–2.75)]. This finding is similar with the same studies conducted in Hadiya Zone [8], and Sidama Zone Dale Woreda [9]. This could be due to the fact that pregnant mother who have an extra meals would have got the chance tp avoid the food they dislike.

Young adult women were more likely to experience food aversion during their pregnancy. About 81.2% of the participants of this study who practiced food aversion were below the age of 33 years. Our study found that, pregnant women of age group 24–28 and 29–33 of years were 3 and 2 times more likely to experience food aversion as compared to pregnant women of age ≥ 34 years [AOR = 3.04, 95% CI (1.72–5.35)] and [AOR = 2.00, 95% CI (1.02–3.92)] respectively.

On the other hand, anthropometric indices that indicate poor maternal nutritional status could predict a decrease in dietary aversions during pregnancy [1]. Accordingly, our study result revealed that, more than one third (34.6%) of the study participants were found to be undernourished (MUAC < 23 cm), which is incomparable to the national prevalence (22%) [24]. The observed high prevalence of undernutrrition might be due to the presence of high prevalence of commonly consumed food aversions in this study among pregnant women, which decrease food choices and, thus, leading to reduced dietary intake, which in turn leads the woman to be undernourished. Furthermore, merely half (49.3%) of the study participants ate three times per day, which is normally recommended for non-pregnant women. Plus to this, about one-fifth (20.5%) of the participants skip their regular meals. Unfortunately, among participants who skept meal, 76% of them experienced food aversion. This implies that, the study participants in this study area obtain suboptimal nutrition during their pregnancy period. As a result, the observed high prevalence of under nutrition among the women could be explained by such overlapping factors. A study report showed that there are positive relationships between nausea and nutritional outcome of a mother [13]. Inline with this evidence our study found that more than half (56.5%) of pregnant women who had nausea were undernourished.

On the other hand, our study revealed that, nutritional status of pregnant women and food aversion have statistically significant association (p-value < 0.001). Meaning that, wellnourished pregnant women had 62% times reduced chance of experiencing food aversion as compared to undernourished pregnant women [AOR = 0.38, 95% CI (0.23–0.62)]. Hence pregnant women who practice food aversion are more likely to be malnourished [13]. This study has supported by a study conducted in Nigeria [7]. This might be due to the observed suboptimal nutrition and meal skipping practice of the study participants. It could also explain that, the study participants commonly avoid the stable foods without devising complement foods which will nourish them. Moreover, it might be due to the avoidance of foods with high nutrient value owing to the fear of having a big baby and enduring a difficult labor [25]. Incontrary to this study, study conducted in Ethiopia reported non-significant statistical association [9]. On the other hand, similar study conducted at Southern Ethiopia revealed none significant association between food aversion and nutritional status of pregnant women [8]. The observed difference could be due to socio-economic variation of the study participants as the economic constraints and intra‐household distribution of food are key barriers to achieve adequate dietary intake during pregnancy [25].

The strength of this study is that it is amongst few community based studies in Ethiopia conducted to assess prevalence of food aversions and its association with nutritional status of pregnant women during pregnancy. Moreover, we have employed a mixed type of study design with relatively high sample size which we believe that it will provide results that can be generalized to the target population. Despite its strength, the limitations of the study could be a potential introduction of a recall bias on some variables as the data were taken through interviews retrospectively and also the study relied on only MUAC for the anthropometric measurement of mothers so as to determine thier nutritional status. Further, by its nature cross sectional design couldn’t determine the cause and effect relationships between food aversion and nutritional status of women. Observational studies are needed to determine temporal relationship of food aversion on nutritional status of pregnant women.

## Conclusion and recommendations

A total of 497 pregnant women were interviewed voluntarily with a response rate of 98.4%. The finding of this study revealed that the overall prevalence of food aversion was relatively high (69.2%) and cereal and enset were the most frequently averted foods. The mean (± SD) MUAC measurement of the study participants was 22.7(± 2.4) cm. Relatively high prevalence of undernutriton (34.6%) among pregnant women was found. Aged group 19–23 and 29–33 years, nausea, and having additional meal during pregnancy were significantly associated with food aversion. Likewise, statistically significant (*p*-value < 0.001) association between nutritional status of pregnant women and food aversion was obtained. Therefore, the Woreda Health Office needs to be intensified on nutritional needs of pregnant women and the implications of food aversion during pregnancy to ensure pregnant women have optimal meal pattern and good nutritional status through strengthening nutrition education for each pregnant woman during rputine ANC visit. Considering incorporation of maternal nutrition into preservice and in‐service curriculums and trainings of health providers and community level workers is aslo critical. Further research concerning the relationship between aversion and nutritoanl status of the pregnant women is also crucial.

## Data Availability

Not applicable.

## Abbreviations

ANC: Ante Natal Care
AOR: Adjusted Odds Ratio
BMI: Body Mass Index
COR: Crude Odds Ratio
DNA: Deoxy-ribo Nucleic Acid
IDD: Iodine Deficiency Disorder
LBW: Low Birth Weight
MUAC: Mid-Upper Arm Circumference
RDI: Recommended Daily Intake
RNA: Ribo Nucleic Acid
SNNPR: South Nation Nationalities and People Region
SPSS: Statistical Package for Social Sciences
WHO: World Health Organization
